# AR-12 Has a Bactericidal Activity and a Synergistic Effect with Gentamicin against Group A *Streptococcus*

**DOI:** 10.3390/ijms222111617

**Published:** 2021-10-27

**Authors:** Nina Tsao, Ya-Chu Chang, Sung-Yuan Hsieh, Tang-Chi Li, Ching-Chen Chiu, Hai-Han Yu, Tzu-Ching Hsu, Chih-Feng Kuo

**Affiliations:** 1 Department of Medical Laboratory Science, College of Medical Science and Technology, I-Shou University, Kaohsiung 824005, Taiwan; ninatsao@isu.edu.tw (N.T.); a0962080882@gmail.com (Y.-C.C.); vicky091888@gmail.com (T.-C.L.); jane76425@gmail.com (C.-C.C.); 2 Department of Biological Science and Technology, College of Medical Science and Technology, I-Shou University, Kaohsiung 824005, Taiwan; yuhaihantaiwan@gmail.com (H.-H.Y.); penny0912832972@gmail.com (T.-C.H.); 3 Bioresource Collection and Research Center, Food Industry Research and Development Institute, Hsinchu 300024, Taiwan; sungyuan@gmail.com; 4 School of Medicine, I-Shou University, Kaohsiung 824005, Taiwan; 5 Department of Nursing, College of Medicine, I-Shou University, Kaohsiung 824005, Taiwan

**Keywords:** AR-12, group A *Streptococcus* (GAS), invasive infection, synergistic effect

## Abstract

*Streptococcus pyogenes* (group A *Streptococcus* (GAS) is an important human pathogen that can cause severe invasive infection, such as necrotizing fasciitis and streptococcal toxic shock syndrome. The mortality rate of streptococcal toxic shock syndrome ranges from 20% to 50% in spite of antibiotics administration. AR-12, a pyrazole derivative, has been reported to inhibit the infection of viruses, intracellular bacteria, and fungi. In this report, we evaluated the bactericidal activities and mechanisms of AR-12 on GAS infection. Our in vitro results showed that AR-12 dose-dependently reduced the GAS growth, and 2.5 μg/mL of AR-12 significantly killed GAS within 2 h. AR-12 caused a remarkable reduction in nucleic acid and protein content of GAS. The expression of heat shock protein DnaK and streptococcal exotoxins was also inhibited by AR-12. Surveys of the GAS architecture by scanning electron microscopy revealed that AR-12-treated GAS displayed incomplete septa and micro-spherical structures protruding out of cell walls. Moreover, the combination of AR-12 and gentamicin had a synergistic antibacterial activity against GAS replication for both in vitro and in vivo infection. Taken together, these novel findings obtained in this study may provide a new therapeutic strategy for invasive GAS infection.

## 1. Introduction

Group A *Streptococcus* (GAS), *Streptococcus pyogenes*, is an important human pathogen, which induces a wide spectrum of symptoms, from superficial skin and throat infections to life-threatening streptococcal toxic shock syndrome and necrotizing soft tissue infections. The mortality rate of the streptococcal toxic shock syndrome may exceed 50% in spite of aggressive treatment [1,2,3]. GAS exhibits global morbidity and mortality, and the estimated incidence of invasive GAS infection is around 2 to 4 per 100,000 people in developed countries and ranges from 12 to 83 per 100,000 people in developing countries [4]. The increasing occurrence of antibiotic resistance in GAS infection may be associated with many significant outbreaks [5,6,7].

AR-12 (OSU-03012), a pyrazole-based compound derived from celecoxib, has completed the first phase of clinical trials as an anticancer agent [8]. AR-12 inhibits the expression of heat shock proteins, plasma membrane receptors, and drug efflux pumps in tumor cells [9]. It can inhibit the activity of protein kinase B, Janus-activated kinase 2 (JAK2), p21-activated kinase (PAK), 3-phosphoinositide dependent kinase 1 (PDK1), phosphatidylinositol 3-kinase (PI3K), signal transducer, and activator of transcription 3 (STAT3), surviving, and X-linked inhibitor of apoptosis (XIAP) of tumor cells [8,10,11,12,13]. In addition, AR-12 activates caspase- and cathepsin-dependent cytotoxicity and is applied to inhibit tumor cells growth in vitro and in vivo [10,11,14]. It also regulates the expression of ATG5, Beclin1, BiP/GRP78, and HSP90 to cause the pressure of the endoplasmic reticulum and induce autophagy of tumor cells [15,16].

Reports also indicate that AR-12 can inhibit fungal acetyl CoA synthase and PDK1 activity, and improve the antifungal activity of fluconazole to inhibit the growth of *Cryptococcus neoformans* [17,18]. In addition, AR-12 can inhibit the replication of the Lassa virus, Ebola virus, Marburg virus, Zika virus, and Dengue virus in cells, increasing the survival rate of virus-infected cells [19,20,21]. AR-12 inhibits the growth of intracellular pathogens, such as *Salmonella enterica*, *Francisella tularensis*, and *Leishmania donovani* [22,23,24]. Zhang et al. indicate that AR-12 inhibits the replication of *Mycobacterium abscessus* in cells but also reduces the bacterial levels in the lungs of *M. abscessus*-infected mice [25]. Celecoxib, a clinical COX-2 inhibitor for the treatment of rheumatoid arthritis and osteoarthritis, has been reported to directly inhibit the growth of *Staphylococcus aureus*, *Streptococcus pneumonia*, *Listeria monocytogenes*, *Bacillus anthracis*, and *Mycobacterium smegmatis* [26]. Several celecoxib-based compounds also express in vitro antibacterial activity. The compound 46, one of the celecoxib derivatives, has a similar chemical structure to AR-12 and shows in vitro and in vivo antibacterial activity against methicillin-resistant *Staphylococcus aureus* [27]. The cytotoxic effect of compound 46 is specific to Gram-positive bacteria [27]. We considered the celecoxib derivative AR-12 may have similar antibacterial activity against Gram-positive GAS. In this study, we demonstrate that AR-12 had bacteriostatic and bactericidal effects against two GAS strains—M1-type A20 and M49-type NZ131. Based on spectrophotometry and colony-forming unit (CFU)-based assay, our in vitro results showed that AR-12 dose-dependently reduced the bacterial growth during the experimental periods, and 2.5 μg/mL of AR-12 significantly reduced the viability of GAS. The bactericidal mechanism of AR-12 was evaluated by flow cytometry and Western blot. The SYPRO- and propidium iodide-staining assays by flow cytometry indicate that the AR-12 treatment decreased the content of nucleic acids and proteins of GAS. To explore whether AR-12 can synergize antibiotics to kill GAS and achieve decreased antibiotic doses, we also examined the effects of a combination of sub-minimum inhibitory concentrations (sub-MICs) of AR-12 and gentamicin against GAS. The results showed the combination of sub-MICs of AR-12 and gentamicin had a synergistic antibacterial activity against both GAS strains. Taken together, the novel findings obtained in this study may provide a new therapeutic strategy against invasive group A *Streptococcus* infection.

## 2. Results

### 2.1. Inhibition of In Vitro GAS Growth by AR-12

**Figure 1:**
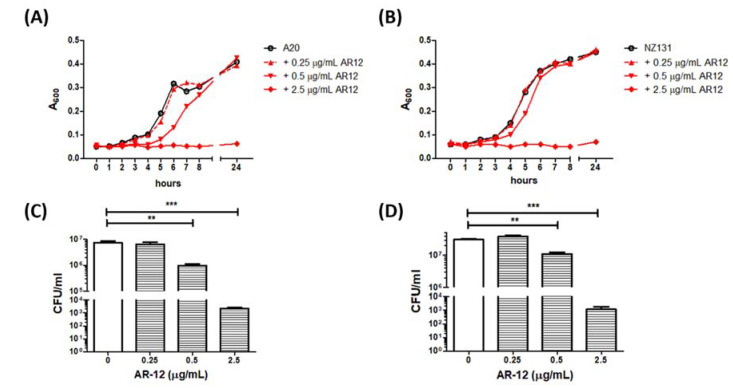
AR-12 inhibited growth of group A *Streptococcus* in vitro. The M1-type GAS A20 strain (**A**,**C**) or the M49-type GAS NZ131 strain (**B**,**D**) was cultured in THY medium with different concentrations of AR-12. The growth curve of GAS after AR-12 treatment on both GAS strains was estimated by spectrophotometry (**A**,**B**) and by CFU-based assay (**C**,**D**). The turbidity of the bacteria was determined with a spectrophotometer by measuring the absorbance at 600 nm (A_600_). The number of remaining bacteria in each group after 2 h cultivation of AR-12 was quantified by plating on THY agar plates. Treatment groups were compared for significance using ANOVA. ** *p* < 0.01, *** *p* < 0.001 compared with the relative groups as shown in the figure.

The results of the spectrophotometric analysis showed that 0.5 μg/mL of AR-12 reduced the growth rates of both GAS strains A20 and NZ131, while 2.5 μg/mL of AR-12 completely inhibited bacterial growth (Figure 1A,B). The bactericidal activity of AR-12 on GAS was further examined by a CFU-based assay at 2 h or 24 h after AR-12 treatment. The cell numbers of surviving A20 and NZ131 strain were reduced from 7.4 × 10^6^ CFU/mL and 3.8 × 10^7^ CFU/mL to 2.3 × 10^3^ CFU/mL and 1.1 × 10^3^ CFU/mL by 2.5 μg/mL of AR-12 treatment for 2 h incubation, respectively. After 24 h incubation with 2.5 μg/mL of AR-12, the remaining bacteria of A20 and NZ131 strains amounted to 5.9 × 10^5^ CFU/mL and 1.2 × 10^5^ CFU/mL, respectively, which were two log folds lower than those of the medium control, 0.25 μg/mL, or 0.5 μg/mL of AR-12-treated groups. These data suggest that AR-12, at a concentration ≥2.5 μg/mL rapidly killed both GAS strains, and 90% of the A20 and NZ131 GAS cells were inhibited by 2.5 μg/mL of AR-12. This indicates that the MIC at 90% inhibition level (MIC_90_) of AR-12 against both GAS strains was 2.5 μg/mL. In addition, compared with the medium-only treatment of A20 cells (7.4 × 10^6^ CFU/mL) and NZ131 cells (3.8 × 10^7^ CFU/mL) at 2 h incubation time, there were 1.0 × 10^6^ CFU/mL of A20 cells and 1.1 × 10^7^ CFU/mL of NZ131 cells after 2 h treatment by 0.5 μg/mL of AR-12, respectively, and this indicates that AR-12 temporary inhibited A20 and NZ131 cell growth at a concentration of 0.5 μg/mL (Figure 1C,D). These results suggest that AR-12 presented both bacteriostatic and bactericidal effects against the two tested GAS strains, M1-type A20, and M49-type NZ131.

### 2.2. AR-12 Reduced the Nucleic Acid and Protein Content of Group A Streptococcus

**Figure 2:**
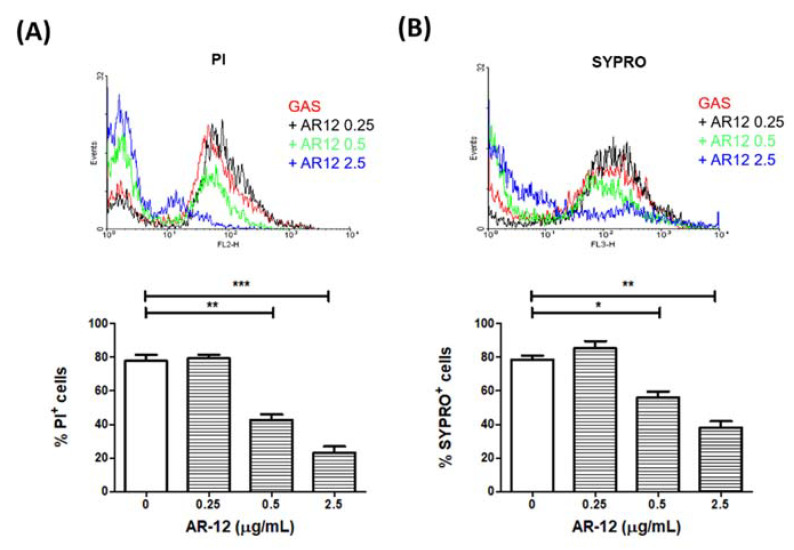
AR-12 reduced the nucleic acid and protein contents of group A *Streptococcus*. The M49-type GAS NZ131 strain was cultured with different concentrations of AR-12 for 2 h. The total protein and nucleic acid content of GAS were stained with propidium iodide dye (**A**) and SYPRO dye (**B**), respectively, and then analyzed by flow cytometer. The result was determined by the mean fluorescence intensity (MFI) of each group. The percentages of PI^+^ or SYPRO^+^ cells were calculated and expressed as the mean ± standard deviation. Treatment groups were compared for significance using ANOVA. * *p* < 0.05, ** *p* < 0.01, *** *p* < 0.001 compared with the relative groups as shown in the figure.

To examine the mechanisms of the bacteriostatic and bactericidal effects of AR-12, the GAS strain NZ131 was incubated with different concentrations of AR-12 for 2 h and fixed with 4% paraformaldehyde. The fixed GAS cells were incubated with SYPRO dye and propidium iodide to analyze the amounts of proteins and nucleic acids of GAS by a flow cytometer, respectively. The results of flow cytometry analysis indicated that there was a reduction of approximately 40% of nucleic acid content, and 25% of the protein content of GAS following 0.5 μg/mL of AR-12 treatment for 2 h, compared with those of the non-treated groups. Moreover, the treatment of 2.5 μg/mL of AR-12 reduced the nucleic acid and protein contents of GAS by 60% and 40%, respectively (Figure 2A,B). These results suggest that AR-12 achieves bacteriostatic and bactericidal activities by interfering with the synthesis of nucleic acids and proteins on GAS.

### 2.3. AR-12 Treatment Reduced the Production of Heat Shock Proteins and Exotoxins by GAS

**Figure 3:**
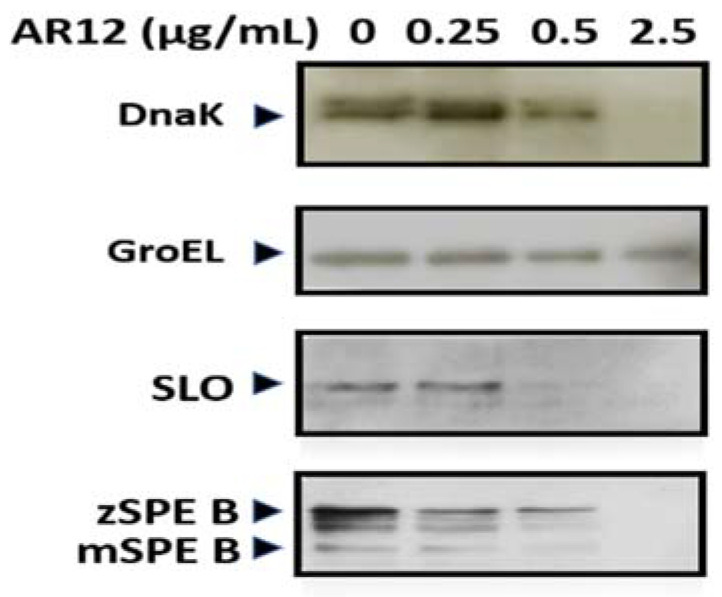
The expression of heat shock proteins and GAS exotoxins by AR-12 treatment. The NZ131 strain was incubated with different concentrations of AR-12 at 37 °C for 2 h, and total protein was isolated by bacterial total protein extraction kit. The protein samples were separated using SDS–PAGE and blotted with anti-DnaK or anti-GroEL antibodies and developed using an ECL Western blot detection kit. Furthermore, the mid-log and late-log phases of the GAS culture supernatants with or without AR-12 treatment were assessed by Western blotting using anti-SPE B and anti-SLO monoclonal antibody, as described in the Materials and Methods Section.

Heat shock proteins are highly conserved in bacteria, and they are utilized against stresses to increase bacterial viability. The cytoplasmic heat shock proteins DnaK and GroEL of the medium-treated or the AR-12-treated GAS were collected at the same incubation time and analyzed by Western blotting. Results indicated that the total amount of DnaK was significantly reduced, but the level of GroEL only slightly decreased, with 0.5 or 2.5 μg/mL of AR-12 (Figure 3). We further tested the exotoxin levels of streptolysin O (SLO) and streptococcal pyrogenic exotoxin B (SPE B) in the culture supernatants, which were collected from medium-only GAS culture or the AR-12-treated GAS culture. The results of Western blotting showed that a significant reduction in SLO was found in the 0.5 μg/mL of AR-12-treated GAS supernatant, while the reduction in the SPE B zymogen (42 kDa) and the mature form of SPE B (28 kDa) was detected in 0.25 μg/mL of AR-12-treated GAS supernatant (Figure 3). These results indicate that the AR-12 treatment inhibited the production of some heat shock proteins and the GAS exotoxins, such as SLO and SPE B.

### 2.4. The Analysis of Scanning Electron Microscopy

**Figure 4:**
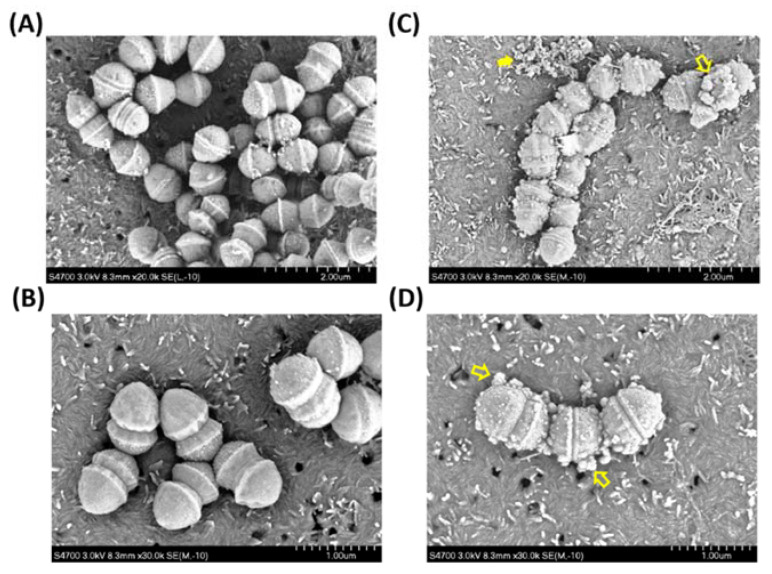
SEM analysis of the AR-12-treated GAS. SEM analysis was performed on NZ131 strain, which was incubated in the absence (**A**,**B**) or the presence of 2.5 μg/mL of AR-12 (**C**,**D**) for 2 h. The untreated GAS showed a smooth cell wall (**A**,**B**), whereas GAS treated with AR-12 exhibited vesicle-like structures dispersed over the cell wall (hollow arrows, (**C**,**D**)) and displayed a large aggregation of vesicle-like structures (solid arrows, (**C**)). Scale bars: 2 μm (**A**,**C**); 1 μm (**B**,**D**).

The scanning electron microscopy (SEM) was further used to analyze the surface morphological change of the AR-12-treated NZ131 strain. After treatment with 2.5 μg/mL of AR-12 for 2 h, the SEM images revealed the remarkable change in GAS architecture. Untreated NZ131 cells showed a smooth surface pattern of cell walls (Figure 4A,B), whereas the NZ131 cells treated with 2.5 μg/mL of AR-12 displayed several micro-spherical structures protruding out of cell walls (Figure 4C,D). The AR-12-treated GAS cells also displayed incomplete septa and a reduction in the bacterial division. By contrast, normal septum formation and bacterial separation appeared in the THY medium control group (Figure 4B). These SEM results suggest that the bactericidal activity of AR-12 might be due to the impedance of the synthesis of proteins and nucleic acids, which further damaged the bacterial surface structures by a weakening of the cell wall of GAS.

### 2.5. The Cell Effects of AR-12

**Figure 5:**
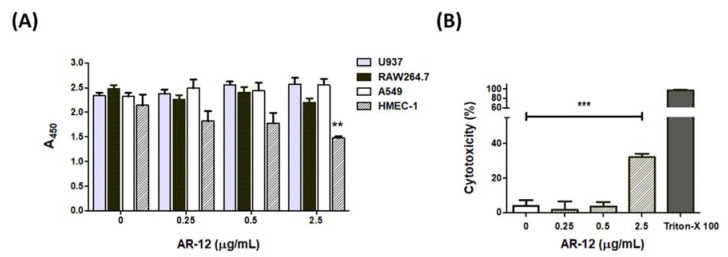
Cytotoxic analysis of AR-12. Cells were incubated with 0.25, 0.5, and 2.5 μg/mL of AR-12 for 24 h: (**A**) The growth of A549, HMEC-1, RAW264.7, or U937 cell lines was determined by the WST-1 colorimetric assay. Absorbance was measured at 450 nm, and the results of three experiments are represented and expressed as the mean ± standard deviation; (**B**) the culture supernatants of HMEC-1 cells in the presence of different concentrations of AR-12 were collected at 24 h post treatment and then measured by the LDH detection kit. The cytotoxicity % was calculated, as described in the Materials and Methods Section. The results of the three experiments are represented and expressed as the mean ± standard deviation. Treatment groups were compared for significance using ANOVA. ** *p* < 0.01, *** *p* < 0.001 compared with the relative groups as shown in the figure.

Reports have indicated that AR-12 inhibits the activity of several protein kinases, including AKT, JAK2, PDK1, and STAT3 of different mammalian cell lines and induces cell death [8,10,11,12,13,14]. We used the WST-1 assay to test whether the bacteriostatic and bactericidal concentrations of AR-12 were cytotoxic to mammalian cell lines. The results of WST-1 analysis indicated that AR-12 exhibited low cytotoxicity in various cell lines, including A549 cells, RAW264.7 cells, and U937 cells at concentrations ≤ 2.5 μg/mL. However, a significant reduction in the absorbance value of the WST-1 assay appeared in the AR-12-treated HMEC-1 cells, suggesting that AR-12 may be cytotoxic to HMEC-1 cells (Figure 5A). WST-1 is a sensitive colorimetric assay for determining cell proliferation or cytotoxicity. However, the amount of formazan dye formed is directly related to the metabolic activity of cells in the WST-1 assay. To investigate whether the bacteriostatic or bactericidal concentrations of AR-12 inhibited cell metabolic activity or directly induced cell death in HMEC-1 cells, we examined the amounts of lactate dehydrogenase released from HMEC-1 cells under various concentrations of AR-12 treatment for 24 h. Results showed there were no statistical differences in LDH release among the medium control group and those of the AR-12-treated A549 cells, RAW264.7 cells, or U937 cells. However, a significant elevation of extracellular LDH level was found in HMEC-1 cells treated with 2.5 μg/mL of AR-12 (Figure 5B), which indicated the bactericidal concentration of AR-12 could directly induce cell death of HMEC-1 cells, while the other tested cell lines were more tolerant to AR-12 treatment. These results indicate that the concentrations of ≤0.5 μg/mL of AR-12 showed non-toxicity to A549, HMEC-1, RAW264.7, and U937 cell lines.

### 2.6. AR-12 and Gentamicin Had a Synergistic Effect against Group A Streptococcus

A previous study indicates that AR-12 can enhance the antibacterial effects of polymyxin B and colistin to kill the multidrug resistant strains of *Acinetobacter baumannii* and *Klebsiella pneumoniae* [28]. In this study, the effect of a combination of AR-12 with gentamicin to act against group A *Streptococcus* A20 strain and NZ131 strain was evaluated. Our previous data indicated that the MIC_90_ of AR-12 against both GAS strains was 2.5 μg/mL (Figure 1). The MIC_90_ of gentamicin against NZ131 and A20 was 5 μg/mL and 25 μg/mL, respectively. We used a combination of a sub-MIC dose of AR-12 with a sub-MIC dose of gentamicin to test whether these two compounds had a synergistic or an additive effect against both GAS strains. The antibacterial activities of AR-12 in combination with gentamicin were assessed using the checkerboard assay and the time–kill test.

**Figure 6:**
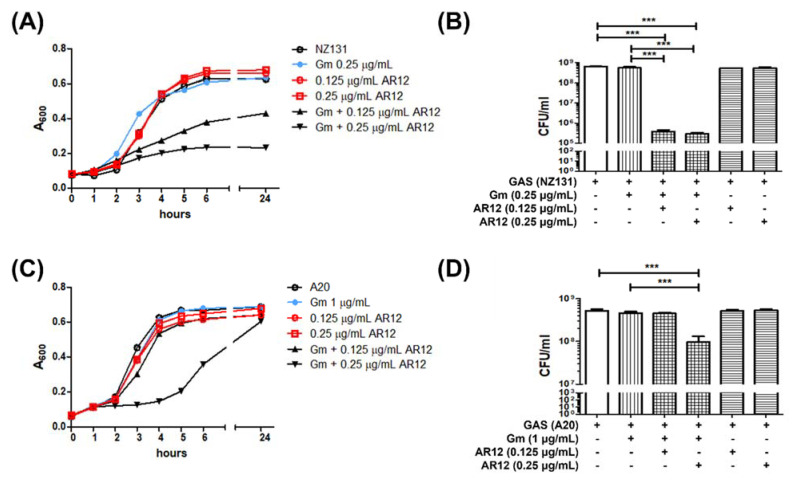
The AR-12 enhanced in vitro antibacterial effect of gentamicin against group A *Streptococcus*. GAS NZ131 cells (**A**,**B**) (5 × 10^6^ CFU/mL) cultured in THY medium were treated with 0.25 μg/mL of gentamicin (Gm), 0.125 or 0.25 μg/mL of AR-12, or combinations of 0.25 μg/mL of Gm and 0.125 μg/mL or 0.25 μg/mL of AR-12. In the experiment of GAS A20 strain influenced by Gm and AR-12, the concentration of Gm was replaced by 1 μg/mL, and concentrations of AR-12 were the same as previously described (**C**,**D**). GAS cultures were incubated at 37 °C, and the A_600_ was measured at various time points over a period of 24 h (**A**,**C**). One of three experiments is represented. The exact bacterial numbers were determined at 4 h by CFU-based assay (**B**,**D**). The results of the three experiments are represented and expressed as the mean ± standard deviation. Treatment groups were compared for significance using ANOVA. *** *p* < 0.001 compared with the relative groups as shown in the figure.

The combination of sub-MICs of AR-12 and gentamicin significantly enhanced the bacteriostatic efficacy beyond each one of them alone. The results of the spectrophotometry assay showed that the growth of the NZ131 strain was significantly inhibited by a combination of 0.125 μg/mL of AR-12 and 0.25 μg/mL of gentamicin (Figure 6A), and the growth of the A20 strain was significantly inhibited by a combination of 0.25 μg/mL of AR-12 and 1 μg/mL of gentamicin (Figure 6C). To confirm the actual bacterial numbers after combination treatment of sub-MICs of gentamicin and AR-12, we used a CFU-based assay to examine the bacterial amount after 4 h treatment. The residual NZ131 number in the combination group, containing 0.125 μg/mL of AR-12 and 0.25 μg/mL of gentamicin, was 3.8 × 10^5^ CFU/mL, which was a significant reduction, compared with that of the gentamicin-only group (5.6 × 10^8^ CFU/mL) or the AR-12-only group (5.2 × 10^8^ CFU/mL) (Figure 6B). However, the combination of 0.25 μg/mL of AR-12 and 1 μg/mL of gentamicin only reduced the A20 number from 4.6 × 10^8^ CFU/mL (the gentamicin-only group) or 5.3 × 10^8^ CFU/mL (the AR-12-only group) to 9.7 × 10^7^ CFU/mL at the same incubation time (Figure 6D). Even though the sub-MIC doses used to inhibit the GAS NZ131 and A20 strains were different, both results of the bacterial growth and the CFU-based assay suggest that AR-12 potentiated the antibacterial efficacy of the sub-MICs of gentamicin.

Based on the equation used to figure out the antibacterial mechanism of compounds [29,30], we calculated the fractional inhibitory concentration (FIC) and the cumulative fractional inhibitory concentration index (ΣFIC) by the following formulae: FIC of AR-12 = (MIC of AR-12 in combination)/(MIC of AR-12 alone), FIC of gentamicin = (MIC of gentamicin in combination)/(MIC of gentamicin alone), and the FIC index (ΣFIC) = FIC of AR-12 + FIC of gentamicin. The ΣFIC index was interpreted as follows: A ΣFIC ≤ 0.5 means synergy; 0.5 < ΣFIC ≤ 1 means additivity; 1 < ΣFIC ≤ 4 means indifferent; ΣFIC: >4 means antagonistic [29]. The combination of gentamicin and AR-12 produced an FIC index value of 0.3 for both GAS strains (Table 1). This represents a synergistic effect of the AR-12-gentamicin combination against both GAS strains.

### 2.7. The Survival Rate in the Mouse Model after Combination Treatment

**Figure 7:**
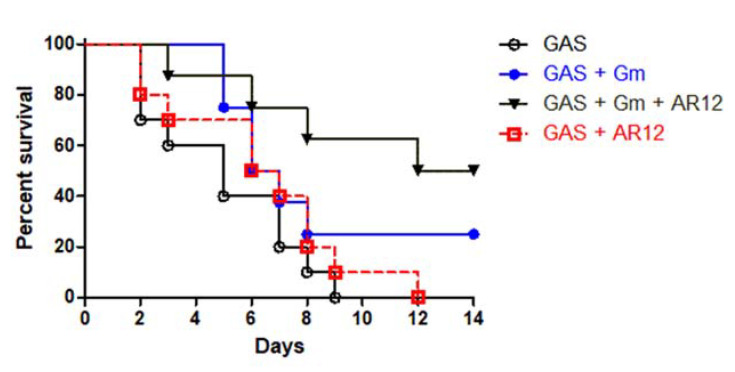
Combination of sub-MICs of gentamicin and AR-12 protected mice from lethal dose of group A streptococcal infection. Groups of eight to ten mice were inoculated via the air pouch route with 2 × 10^8^ CFU of GAS NZ131 strain. After the GAS inoculation, an air pouch injection of 2 μg/kg of gentamicin, 10 μg/kg of AR-12, or the same dosage of gentamicin-AR-12 combination was administered immediately post GAS infection. The animals were observed every day for a total of 14 days. The survival curves were compared for significance using the log-rank test for the drugs treatment groups versus the GAS-infected mice (*p* < 0.01).

We performed an air-pouch GAS infection model to assess in vivo protective efficacy of AR-12. The sub-clinical dosages of AR-12 were examined in the air-pouch infection model [31]. After intra-air pouch injection of 2 × 10^8^ CFU of NZ131, different doses (10 μg/kg to 400 μg/kg) of AR-12 were injected into the air-pouch immediately, and then the survival rates of mice were examined. The results showed that while all of the PBS-treated control mice, the 10 μg/kg and the 100 μg/kg AR-12-treated mice died within 10 days post infection, 10% of the 400 μg/kg AR-12-treated mice survived over the 14-day experimental period. However, there was no statistically significant difference between the AR-12 (400 μg/kg)-treated mice and the GAS-infected control mice. Subsequently, the protective efficacy of the combination of gentamicin and AR-12 was examined. As shown in Figure 7, GAS NZ131 cells (2 × 10^8^ CFU/mouse) without treatment caused 100% of mice death within 9 days; treatment of AR-12 (10 μg/kg) still caused 100% of mice death within 12 days. Treatment of gentamicin (2 μg/kg) with GAS-infected mice caused 25% of mice survival over 14 days, even though there was no statistical difference compared with the GAS-infected group. However, combination treatment of 2 μg/kg of gentamicin and 10 μg/kg of AR-12 significantly elevated the GAS-infected mice survival rate to 50% (*p* < 0.01, Figure 7). These results suggest that non-toxic doses of AR-12 can enhance the in vivo antibacterial effect of gentamicin against GAS infection.

## 3. Discussion

In this study, AR-12, a celecoxib derivative compound, was found to have bacteriostatic and bactericidal effects against GAS (Figure 1). Results showed 2.5 μg/mL of AR-12 made three log folds of viable bacteria decreasing within 2 h, which indicated that AR-12 had a bactericidal effect against GAS. However, the number of viable GAS was maintained at the inoculated concentration of 10^6^ CFU/mL or slightly increased to 10^7^ CFU/mL after 2 h cultivation with 0.5 μg/mL of AR-12. This result indicated the low concentration of AR-12 has no bactericidal but only bacteriostatic effect on GAS. Our data suggest that AR-12, at a concentration ≥2.5 μg/mL, rapidly killed both GAS strains, and 90% of the A20 and NZ131 GAS cells were inhibited by 2.5 μg/mL of AR-12. The antibacterial effect of AR-12 on GAS was also demonstrated by reducing GAS protein and nucleic acid content (Figure 2) and further interfered with GAS replication. Heat shock proteins are highly conserved in bacteria, and they are utilized against stresses and increase bacterial viability. DnaK is a drug target, in the treatment of cancer, viral and bacterial infections, which participates in the folding, activity regulation, and membrane transporting of newly synthesized proteins [32]. Lemos et al. have reported that downregulation of heat shock protein DnaK of *Streptococcus mutans* decreases bacterial growth and impairs biofilm formation [33]. Our Western blot results showed that AR-12 decreased the heat shock protein DnaK levels, which might delay the growth of GAS (Figure 3). Although AR-12 reduced the bacteria amount of GAS, we extracted the bacterial proteins and loaded a total of 20 μg of proteins for each sample for the Western blot assay. Based on the Western blot result of GroEL staining, we considered that Dnak was clearly inhibited by AR-12 but not by inappropriate quantification of studied proteins. Furthermore, our Western blotting results also showed a significantly reduced production of GAS’s virulence factors, streptococcal exotoxins SLO and SPE B, in AR-12-treated GAS supernatants (Figure 3). These results suggest that AR-12 treatment affected GAS growth and also interfered with the release of its virulence factors.

Morphological alteration of the AR-12-treated GAS was confirmed by SEM analysis. SEM results showed the GAS cultured in THY medium appeared with normal septum formation and bacterial division, but after 2 h of AR-12 treatment, the GAS displayed incomplete septa and spherical vesicle-like structures protruding out of the GAS surface (Figure 4C,D). Moreover, the spherical vesicles released from the GAS surface, and large aggregations of spherical structures were observed in the AR-12-treated GAS but not in the medium control GAS. The diameters of those spherical structures ranged from 100 nm to 200 nm, which were similar to the extracellular membrane-derived vesicles of the LL-37-treated GAS. The vesicles derived from LL-37-treated GAS contain several streptococcal virulence factors and induce the inflammatory response, which may be involved in the pathogenesis of GAS [34]. Even though AR-12-induced spherical vesicles released from GAS were similar to vesicles released from LL-37-treated GAS in morphology, we found the protein contents, including heat shock protein DnaK and streptococcal exotoxins SLO and SPE B, were significantly inhibited in AR-12-treated GAS (Figure 2 and Figure 3). We considered that vesicle-like structures were caused by AR-12-mediated weakening of cell walls and led to leakage of cell contents from the GAS cells. Decreased production of GAS virulence factors may impair GAS-induced inflammatory response. Hence, the role of the AR-12-induced release of GAS vesicles needs further investigation.

Both the results of the bacterial growth and the CFU-based assay suggest that a sub-MIC dose of AR-12 potentiated the killing efficacy of a sub-MIC dose of gentamicin and indicate that there was a synergistic effect of the AR-12 and gentamicin to act against GAS. A three log-fold increase in NZ131 killing was observed by 0.125 μg/mL of AR-12 and 0.25 μg/mL of gentamicin combination (Figure 6B). However, the combination of 0.25 μg/mL of AR-12 and 1 μg/mL of gentamicin had poorer efficiency against the A20 strain than that of combination treatment in NZ131 cells. The residual A20 bacterial number of the AR-12-gentamicin combination group was just a fourfold decrease, compared with that of the AR-12-only or gentamicin-only groups. The sub-MICs of AR-12 reduced the MIC of gentamicin required to act against the NZ131 strain and the A20 strain from 1 μg/mL to 0.25 μg/mL and from 5 μg/mL to 1 μg/mL, respectively. Moreover, the sub-MICs of gentamicin also enhanced the antibacterial activity of AR-12, and the MIC of AR-12 against both GAS strains changed from 2.5 μg/mL to 0.25 μg/mL. These results indicate that the combination of sub-MICs of AR-12 synergized with sub-MICs of gentamicin to kill both GAS strains.

Preclinical studies indicate that AR-12 has antitumor activity, and the safety data from a phase-1 clinical trial suggest the recommended phase II dose of AR-12 is 10 mg/kg. Our in vivo model indicated that 0.4 mg/kg of AR-12 treatment only slightly enhanced the survival rate of the GAS-infected mice. However, combinative treatment of the 2 μg/kg of gentamicin and 10 μg/kg of AR-12 significantly increased the GAS-infected mice survival rate (Figure 7). The administrative dose of gentamicin and AR-12 used here was much less than the daily recommended dose of gentamicin (3–6 mg/kg) and AR-12 (10 mg/kg), respectively. Although our WST-1 and LDH assays observed a cytotoxic effect of the 2.5 μg/mL of AR-12-treated HMEC-1 cells, our in vivo gentamicin–AR-12 combinative dosage of AR-12 was 10 folds less than the in vitro dosage.

Previous studies indicate that AR-12 has no direct inhibitory effect on the growth of Gram-negative *Salmonella enterica* serovar Typhimurium, *Francisella novicida, Acinetobacter baumannii*, *Escherichia coli,* and *Klebsiella pneumoniae* [22,23,28]. In this study, we found that the AR-12 reduced the Gram-positive GAS heat shock protein DnaK and delayed GAS replication or directly killed GAS. Bacterial DnaK regulates DNA replication enzyme RecA activity and plays an essential role in bacterial DNA repair and survivability [35]. The results of our flow cytometry analysis indicated that AR-12 reduced the protein and nucleic acid contents of GAS, which suggests that AR-12 achieved bacteriostatic and bactericidal activities through interfering with the synthesis of macromolecules of GAS. AR-12 is a synthetic compound derived from the selective COX-2 inhibitor celecoxib. Celecoxib can block the bacterial multidrug resistance efflux pump and increase the sensitivity of the multidrug resistance of *Staphylococcus aureus* to antibiotics [36]. AR-12 may have an inhibitory efflux pump activity similar to that of celecoxib and, therefore, also has the ability to enhance the antibacterial efficacy of gentamicin. The antibacterial mechanism of gentamicin is through the blocking of bacterial protein synthesis by irreversibly binding to the 30S ribosomal subunits. The synergistic inhibition mechanism of the AR-12–gentamicin combination against GAS needs further examination. This study suggests a new therapeutic strategy against GAS infection with the sub-MIC dosages of gentamicin and AR-12. Based on this study, we hope the treatment by a combination of AR-12 and antibiotics would substantially decrease antibiotic use and antibiotic resistance of bacteria in the future.

## 4. Materials and Methods

### 4.1. Bacteria

GAS strain A20 (M1 serotype) was isolated from a patient with necrotizing fasciitis [37]. GAS strain NZ131 (M49 serotype) was a gift from D. R. Martin, New Zealand Communicable Disease Center, Porirua. The GAS was grown in Todd–Hewitt medium supplemented with 0.2% yeast extract (THY) (Difco Laboratories, Franklin Lakes, NJ, USA) for 12 h at 37 °C and then subcultured into a fresh broth (1:20 (vol/vol)) for another 5 h to obtain the log-phase bacteria. The bacterial density was determined by measuring the absorbance at 600 nm (A_600_) with a spectrophotometer (Beckman Instruments, Brea, CA, USA). The bacterial suspension was diluted with phosphate-buffered saline (PBS) to 2 × 10^9^ CFU/mL for in vivo experiment. To quantitate the exact bacterial concentration, the bacterial suspension was serially diluted with sterile PBS, then 0.1 mL was poured on THY agar plates and incubated at 37 °C overnight.

### 4.2. Bacterial Growth Curves Assay and Evaluation of Antibacterial Concentrations of AR-12 and Gentamicin

A fresh GAS colony was grown in THY broth (Difco Laboratories) for 12 h at 37 °C and then subcultured into the fresh broth. At the time of subculture, varying concentrations of AR-12 or gentamicin were added to the bacterial suspension, and bacterial growth at different time points was determined with a spectrophotometer by measuring A_600_. The MIC of AR-12 or gentamicin was determined by the agar dilution method. For exact quantification of bacterial counts at different time points, the bacterial suspensions were serially diluted, plated on THY agar, and cultured at 37 °C overnight. The results of one of three experiments are reported.

### 4.3. Cell Lines and Cell Culture

A549 and RAW264.7 cell lines were grown in DMEM (Life Technologies, Grand Island, NY, USA) supplemented with 5% fetal bovine serum, 100 μg/mL of penicillin, and 100 μg/mL of streptomycin (Gibco). U937 cells were grown in RPMI 1640 (Life Technologies) supplemented with 5% fetal bovine serum, 100 μg/mL of penicillin, and 100 μg/mL of streptomycin (Gibco). HMEC-1 cells were grown in M200 (Life Technologies) supplemented with low serum growth factor, 100 μg/mL of penicillin, and 100 μg/mL of streptomycin (Gibco, New York, NY, USA). The cells were cultured at 37 °C and with 5% CO_2_ in an incubator (Thermo Fisher Scientific, San Francisco, CA, USA).

### 4.4. Flow Cytometry Assay

To examine the mechanisms of the bacteriostatic and bactericidal effects of AR-12, GAS NZ131 cells were incubated with different concentrations of AR-12 for 2 h and fixed with 4% paraformaldehyde. The fixed bacteria were reacted with SYPRO dye (Thermo Fisher Scientific) in 0.04% SDS for 0.5 h. Then, the fixed GAS was reacted with propidium iodide (PI) dye (Sigma-Aldrich, Saint Louis, MO, USA) for 1 h to analyze the amounts of nucleic acids of GAS. After washing, the bacteria were suspended in 0.5 mL of PBS and analyzed by the flow cytometer FACSCalibur (Becton Dickinson, Franklin Lakes, NJ, USA). The total protein and nucleic acid contents in the GAS were detected by the flow cytometer and determined by the mean fluorescence intensity (MFI) of each group. The percentages of PI^+^ or SYPRO^+^ cells were calculated and expressed as the mean ± standard deviation.

### 4.5. WST-1 Assay

WST-1 is a sensitive colorimetric assay for determining cell viability in cell proliferation and cytotoxicity assays. An appropriate number of cells were plated in 96-well microtiter plates and then treated with 0.25, 0.5, and 2.5 μg/mL of AR-12 for various time courses. The WST-1 reagent solution (Roche) was added to each well of the 96-well microplate containing 100 μL of cells in the culture medium, and the plate was then incubated for 2 h at 37 °C. Absorbance was measured at 450 nm by using a microplate reader (Life Technologies). The results of the three experiments are represented and expressed as the mean ± standard deviation.

### 4.6. LDH Assay

Cell lines were cultivated in the absence or presence of different concentrations of AR-12 at 37 °C and 5% CO_2_ for 24 h, and then the culture supernatants were collected. Cytotoxicity of the AR-12-treated cells was determined using the lactate dehydrogenase (LDH) assay and detected by a microplate reader (Life Technologies). The levels of LDH released into the cell culture supernatants were measured using the LDH Cytotoxicity Detection Kit (Promega, Madison, WI, USA) according to the manufacturer’s instructions. The cytotoxicity percentage was calculated as follows: cytotoxicity % = (experimental group − medium background)/(Triton X − 100 − treated group − medium background) × 100%. The results of the three experiments are represented and expressed as the mean ± standard deviation.

### 4.7. Western Blotting

The NZ131 strain was incubated with different concentrations of AR-12 at 37 °C for 2 h and then harvested by centrifugation (6000 rpm) at 4 °C. After centrifugation, the bacteria were washed twice with ice-cold sterile water and suspended in Tris-EDTA buffer (100 mM of Tris-HCl, 1 mM EDTA, 25% glucose) containing 200 μL of lysozyme (20 mg/mL; Sigma-Aldrich) and 50 μL of mutanolysin (5000 U/mL; Sigma-Aldrich). Total protein contents of each group were isolated using the Bacterial Total Protein Extraction Kit (Invent Biotechnology, Plymouth, MN, USA). Then, 20 μg of the protein samples were separated using SDS–PAGE and then transferred to the PVDF membrane. After blocking, the blots were hybridized with anti-DnaK or anti-GroEL antibodies as the primary antibody, and horseradish peroxidase-conjugated goat anti-rabbit IgG (Cell signaling Technology, Danvers, MA, USA) was used as the secondary antibody. Finally, blots were developed using an ECL Western Blot Detection Kit (Merck-Millipore, Darmstadt, Germany) according to the manufacturer’s instructions. In addition, streptolysin O (SLO) and streptococcal pyrogenic exotoxin B (SPE B) were collected from the mid-log (A_600_ = 0.2~0.3) and late-log (A_600_ = 0.4–0.5) phases of the GAS culture supernatants, respectively, and these culture supernatants were incubated with different concentrations of AR-12. Subsequently, these supernatants were confirmed by Western blot analysis using anti-SPE B and anti-SLO monoclonal antibodies (Sigma-Aldrich). The horseradish peroxidase-conjugated goat anti-mouse IgG (Cell signaling Technology) was used as the secondary antibody, and the blots were developed by ECL, as previously described.

### 4.8. Scanning Electron Microscopy Analysis

GAS NZ131 cells were grown in THY medium in the absence or presence of 2.5 μg/mL of AR-12 for 2 h. After treatment, 0.2 mL of bacteria was dropped onto the Whatman Nuclepore Membrane (Merck-Sigma Aldrich, Darmstadt, Germany) and fixed with 1% aqueous osmium tetroxide in 0.1 M PBS solution at 4 °C for 24 h. The fixed GAS was washed three times in 0.1 M PBS, serially dehydrated from 10 to 100% ethanol, followed by 33%, 66%, and absolute acetone, and dried in a Hitachi HCP-2 critical point dryer. The specimens were mounted and coated with gold by Hitachi E-1045 ion sputter and investigated with a Hitachi S-4700 scanning electron microscope at 1 kV.

### 4.9. Evaluation of Drug Effects Using the Air Pouch GAS Infection Model

BALB/c mice were purchased from the National Laboratory Animal Center in Taiwan. Eight-week-old male mice (body weight 25 ± 1.5 g per mouse) were used in all experiments. The animals were raised and cared for in accordance with guidelines established by the Ministry of Science and Technology in Taiwan. All procedures, care, and handling of the animals were reviewed and approved by the Institutional Animal Care and Use Committee at I-Shou University (ISU108022). Groups of eight to ten mice were anesthetized and then injected subcutaneously with 1 mL to form an air pouch. The NZ131 cell suspension containing 2 × 10^8^ CFU in 0.1 mL of sterile PBS was inoculated into the air pouch. After the GAS inoculation, 0.1 mL of different doses of AR-12 (10, 100, or 400 μg/kg) was given immediately into the air pouch. To determine the effects of the combination of AR-12 and gentamicin on GAS infection, 2 μg/kg of gentamicin, 10 μg/kg of AR-12, and a mixture of both agents were administered immediately post GAS infection. The animals were observed every day, and the mice survival rate was examined for a total of 14 days.

### 4.10. Statistics

The statistical analysis was conducted using Prism 5.0 software (GraphPad Software, San Diego, CA, USA). The data shown in Figure 1, Figure 2, Figure 5 and Figure 6 were compared using one-way ANOVA, and significant differences were evaluated by Tukey’s test for multiple comparisons. For the mouse survival in Figure 7, survival curves were compared for significance using the log-rank test. Statistical significance was set at * *p* < 0.05, ** *p* < 0.01, and *** *p* < 0.001.

## 5. Conclusions

In this study, we demonstrated that AR-12 has an anti-GAS effect in vitro and in vivo. Moreover, the mechanisms for the antibacterial effect of AR-12 on the GAS strain NZ131 cells were evaluated, leading to the indication that AR-12 impeded the synthesis of proteins and nucleic acids, and damaged the bacterial surface structures by weakening the cell wall of GAS. Furthermore, there was a synergistic effect of the AR-12 and gentamicin to act against GAS. The sub-MIC dose of AR-12 potentiated the GAS killing efficacy of a sub-MIC dose of gentamicin in vitro and in vivo. Thus, these results suggest the possible pharmacological use of AR-12 as an effective anti-GAS agent and may provide a new therapeutic strategy for invasive GAS infection.

## Figures and Tables

**Table 1:** AR-12 enhanced the in vitro antibacterial activity of gentamicin against group A *Streptococcus*.

GAS Strain	NZ131 (M49)	A20 (M1)
MIC ranges of Gm (μg/mL)	1–5	5–25
MIC ranges of AR-12 (μg/mL)	1–2.5	1–2.5
MIC_90_ of Gm (μg/mL)	5	25
MIC_90_ of AR-12 (μg/mL)	2.5	2.5
FICGm	0.25	0.2
FICAR-12	0.05	0.1
ΣFIC index	0.3	0.3

Gm: gentamicin.

## Data Availability

The datasets generated during and/or analyzed during the current study are available from the corresponding author on reasonable request.
